
JOURNAL INFORMATION
==============================
NLM Title Abbreviation: Med Chir Rev
NLM Journal ID: 0034617

The Medico-Chirurgical Review

Publisher: Biomedical Journal Digitization Project

ARTICLE INFORMATION
==============================
PMCID: PMC4966187
PMID: 29917609
Article version: 1
Subjects: Reviews

Dr. Marshall on Feigned Diseases

Print publication date: 1828 Jul 1
Volume: 9
Issue: 17
First page: 90
Last page: 96
1828 May 24
License: This work is free of known copyright restrictions. For more information, please see PMC Back Issue Digitization.
License URL: https://creativecommons.org/publicdomain/mark/1.0/

==============================

*or derangement of parts, can possibly occur.* There is no vacillation, no permanent alteration of form. The *flexions* and *flexures* are performed with ease. These motions having ceased, and the column being again at rest, the parts return to their former places. When, however, a particular attitude is frequently repeated, or long persevered in, the ligaments concerned gradually accommodate themselves to the acquired position. Should this constrained habit be much longer continued, deformity will assuredly supervene. Such is, I conceive, the origin and progress of spinal complaints, when they proceed from weakness in the ligaments. In your case the disorder seems to have commenced in them,' and can, I think, be removed by restoring the extruded vertebræ to their former stations. With a view to effect this desirable change, I propose to employ strong frictions to the prominent vertebræ. These to be followed by well regulated pressure, directed to the same parts. —After the process has concluded, I should confine the vertebræ with long slips of adhesive plaster, to prevent their receding. Lastly, the trunk is to be immediately replaced in the supine direction, and to be furnished with a mechanical contrivance to maintain constant pressure upon the prominent vertebræ. These are some of the means, which I venture to suggest, for the cure of your malady. As the vertebræ project almost straight backwards, I think the proposed plan is more likely to succeed, than if they had taken a lateral, or forward direction. I think it only candid in me to inform you, that after a diligent search among the medical books in my possession, I can no where discover any authority either in support of the doctrine, or the practice, that I have ventured to advocate."

After this specimen of the "bolder flight" which Dr. Harrison has taken from the low ranks of his brethren, to the GENERAL READER, we must lay down the pen. We are positively ashamed (if it will be believed that a REVIEWER can have any sense of shame) to record such a passage from the writings of a physician of the present period, on our pages. But the above extract will characterise the book under review, better than any thing which we could say. The pathology is erroneous—the practice is, for the most part, concealed—and the plates are more calculated to frighten the GENERAL READER than to convey any information to the MEDICAL PRACTITIONER.

---

### X.

*Hints to young Medical Officers of the Army on the Examination of Recruits, and respecting the feigned Disabilities of Soldiers; with Official Documents, and the Regulations for the Inspection of Conscripts for the French and Prussian Armies.* By HENRY MARSHALL, Surgeon to the Forces. Octavo, 224. London, 1828.

THAT portion of Forensic or Judicial Medicine which more particularly belongs to the military service of the country, we believe, has not hitherto been treated, by any person, in a distinct work. We are aware that the subject has been incidentally touched by several authors of the highest repute, viz. Dr. Gordon Smith, Messrs. Paris and Fonblanque, Dr. Beck, and particularly Dr. Hennen, in his "Principles of Military Surgery." But, it has long been a desirable object that the military surgeon should be put into possession of a portable work, to serve him as a manual, upon the feigned defects and disabilities of recruits and soldiers, apart from the extensive range of medical jurisprudence, taken in the valuable works above-mentioned, and which, from their very nature, are too voluminous for the library of a military medical officer.

It appears to be the object of Mr. Marshall to bring before the view of his junior brethren, in a concentrated shape, the most approved method of examining recruits, an enumeration and estimate of those defects which are considered to incapacitate, or, to render a man unfit for military service, and also, to give an account of the defects or diseases most commonly assumed by the malingering soldier to avoid his duty, or to escape from the service. The author has very judiciously led the way to these intentions, by prefixing certain official documents from the War Office, and from the Director General of the Army Medical Department. He has also given the regulations for the "Inspection of Conscripts for the French and Prussian Armies," and thus brought into one view the recruiting systems of three of the great military powers of Europe. The following excellent remarks, by the Chief of the Medical Department, stand as a preface to the Prussian Regulations.

"The duty of inspecting recruits, and of determining whether they are fit or unfit for the military service of the country, is one of the most difficult and responsible an army surgeon has to perform. To enable him to execute it correctly and with suitable promptitude, he would require more knowledge and experience than is generally believed; he must possess an intimate acquaintance with anatomy, physiology, and pathology. A knowledge of these sciences is essentially required to qualify him to decide upon the health and general efficiency of recruits, and to distinguish between defects that may be real from those that are only feigned.

"He must also be well acquainted with the duties of the different classes of soldiers, infantry, artillery, and cavalry, during war as well as in peace. The qualifications for performing so important a duty can be acquired only by long servitude and much experience.

"It is impossible to frame specific rules for the examination of recruits, so as to obviate every difficulty. In a great variety of cases the decision must depend on the discretion and experience of the inspecting medical officer."

From our own knowledge of the subject we can add, that in addition to the intimate acquaintance with the above divisions of professional science considered to be requisite by the Prussian Chief, that an experience of the "good and bad qualities of soldiers," as well as a forbearing temper, assiduous attention, and unremitting patience, are qualifications in the medical officer, equally desirable, and, even when possessed in the highest degree, they are not always successful in the detection of fraud. It is not our intention to raise a question, and to decide upon the comparative merits of the regulations for the different services here brought forward; they appear to us to be excellent, and each code to be well adapted for the nation and people for whom it was intended. There is a circumstance, however, in the French regulations, which we notice, because it goes to prove the severity of the conscript system, the rigid manner in which it was enforced, and the almost impossibility of escaping it. They divide the incapabilities for military service into two tables, The first contains "*Evident infirmities, implying absolute incapability of military service*, and which *are left to the municipal administration of the canton.*" The second table "*Infirmities or diseases, which occasion absolute or relative incapability for military service, and which are reserved* for *the examination and opinion of the* central administration of the *department.*" And upon these *relative infirmities* the Code remarks—

"It is difficult to judge readily in every case, which does not present some sensible appearance of an organical lesion. To give a negative judgment would not be just, because the conscript, at the time of his visit, might not find himself in that state which he complains of. On the other hand, he might feign deafness, pains, even a fit of epilepsy, without being in reality subject to any of these diseases, and an exception decided upon so equivocal a ground would be really an infraction of the law. It is therefore necessary to watch these young people, *either in a military hospital or in their private life.* The testimony of the surgeons who attend them, and that of *ten householders* of known probity, who are not related to him, public notoriety, certified by the constituted authorities, are so many means, which, added to recognised rational signs, may increase the probability almost to certainty, and found an impartial judgment. Moreover, as most of these diseases yield to time or skill, there is no need for granting an absolute or definite exemption for those recruits who are afflicted with them, before the surgeons can pronounce their opinion with perfect certainty: it is necessary that the young men should present themselves for examination at stated periods, and sometimes for several months."

Mr. Marshall divides infirmities or defects which disqualify for military service into three classes, viz.

1. Obvious defects (chiefly external.)
2. Defects not obvious (chiefly internal.)
3. Feigned defects.

The obvious defects are mostly enumerated in a small code of "Rules to be observed on the Examination of a Recruit," drawn up for the guidance of district surgeons by the Army Medical Board. We need not repeat them here, but we shall give the author's plan of examination entire, as it appears to us to be perfect; and we apologize for so doing on the score of utility, as it may be useful to civil practitioners who are occasionally called upon to *pass* a recruit, and who may not obtain possession of the work.

"The recruits to be examined are to 'fall in,' and be inspected in their clothes. During this inspection we frequently succeed in detecting deserters, and men who have been in the army, and discharged in consequence of disease and disability. Let them next be examined singly undressed. Upon entering the inspection room, each recruit is to walk a few times pretty smartly across the apartment, for the purpose of ascertaining that he has the perfect use of his inferior extremities. This is a very essential part of the business of inspection. Notwithstanding a rigorous observance of it, however, I have known a medical officer called upon to explain why he approved of a recruit, who, after joining the corps to which he belonged, did not perform the 'goose step' to the entire satisfaction of his commanding officer. He is then to be halted, set up in the position of a soldier under arms, with the knees about an inch apart, and examined from head to foot. The inspection may be conducted with reference to the following qualities, or conditions of the body.

"Colour.

"Muscular capability.

"General health.

"The condition of the external surface, comprehending chronic eruptions, marks of punishment, ulcers, cicatrices, &c.

"The configuration of the thorax, spine, and pelvis.

"The condition of the superior extremities, comprehending symmetry, fractures, contractions, mutilations, &c.

"The condition of the inferior extremities, including symmetry, &c. as also varicose veins, nodes, flatness of the soles of the feet, distorted and supernumerary toes.

"Should no material defect be perceived during this survey, the examination should go on. The recruit is then to perform, in imitation of the hospital sergeant, the following manual evolutions. To stretch out the arms at right angles with the trunk of the body, then touch the shoulders with the fingers, next place the backs of the hands together above the head; in this position let him cough, while at the same time the examiner's hand is applied to the rings of the external oblique muscles. Examine the spermatic chords and testes, then pass the hand over the bones of the legs. The recruit will next stand upon one foot, and move the ancle joint of each extremity alternately. And when any doubt is entertained, respecting the efficiency of the ancle-joint, or any part of an inferior extremity, he should be made to test his strength in that respect by hopping upon the suspected limb for a short period, and the size and aspect of the corresponding joint or part of the opposite limb should also be accurately compared. Let him then extend the superior extremities forward, for the purpose of having his arms and hands examined; he is in this position to perform flexion and extension of the fingers, and to rotate the fore-arms. The head is next to be examined, including the ears, eyes, nose, mouth; then ascertain that he possesses the function of hearing, and the power of distinct utterance; next inquire whether he has passed through the small-pox or been vaccinated. The examination of a recruit in this manner will require about five or six minutes; and if carefully performed, very few disqualified men will be admitted into the service."

"The leading external characters of a good constitution may be briefly enumerated: a tolerably just proportion between the different parts of the trunk and members, a well shaped head, thick hair, a countenance expressive of health, with a lively eye, skin not too white, lips red, teeth white and in good condition, voice strong, skin firm, chest well formed, belly lank, parts of generation well developed, limbs muscular, feet arched, and of a moderate length, hands large."

To look for a specimen of the *beau ideal* of the Greeks, or what may be termed the perfection of beauty in mankind, would appear to be a hopeless search in these kingdoms: for, according to Mr. Marshall,

"Perfect symmetry of the human body may be said never to exist. In almost every individual, a want of harmony in some part or other is discoverable. There is often great want of symmetry between the trunk and inferior extremities, the former being full in proportion to the latter, and vice versa. Even the lateral sections of the body are often disproportioned in size, and not strictly similar in form."*

Mr. Marshall's 2d class, defects not obvious, is chiefly a catalogue of internal disqualifying diseases, such as, according to the French regulations, would not be decided upon as rendering a man totally unfit; but would cause the individuals to be remanded and brought forward, from time to time, until the complaint ceases, or, what would be considered sufficient proofs of its existence are produced. We shall therefore pass on to the 3d class—feigned defects. And here we cannot help remarking, that the author seems to be perfectly at home, he treats the subject *con amore*, he seems to possess, if we may so express it, the most determined antipathy to malingerers of every species. Not contented with explaining their manœuvres in every disease that it is possible to simulate, but in a great many, that it might be thought almost impossible; he publishes their cases without any compunction, and some whom he appears

---

* The following note from page 75, as a curiosity is worthy of attention.

"Abstract of the measurement of the arms and legs of one hundred recruits, which was made with the view of ascertaining the relative equilibrium of the extremities. The arms were measured about equal distances from the shoulder and the elbow, and the legs round the thickest part.

| | Right arm thicker than left. | Left thicker than right. | No difference. | Total. |
|---|---|---|---|---|
| Right handed individuals | 68 | 5 | 18 | 91 |
| Left handed | 1 | 6 | 2 | 9 |
| | | | | 100 |

| No. | Right leg thicker. | Left ditto. | No difference. |
|---|---|---|---|
| 100 | 35 | 37 | 28 |

The difference in the thickness of the limbs was from six eighths of an inch to one eighth.

to have pursued, for a time, that would have disheartened many, having brought to bay, he produces to his readers, with a satisfaction that can only be the result of a conscious performance of duty.

We entirely agree with the author, and every person of experience must, that nothing can be more detrimental to the service or subversive of discipline than a disposition to malingering, and we consider it a serious part of the duty of every military medical officer to check it, and to endeavour to defeat the purpose of the malingerer by the exertion of all his ability, and by every means in his power. These means are very extensive, but like every department of the medical profession, they are not to be acquired without thought and study —there is no royal road to this acquisition any more than to the knowledge of anatomy or pathology. A person may have received the best education that the schools of London, Edinburgh, or Dublin, can grant, and he may be, from his knowledge of diseases, and his method of treating them, perfectly fit to practice among the higher classes of society in the metropolis, and yet (quoad the treatment of simulators) he may be very inefficient for the charge of a regimental hospital. There is, it must be confessed, an irksomeness in the investigation of feigned diseases, that the high bred practitioner, or one just arrived from the schools, might find a difficulty of enduring, and nothing but zeal for the profession and the service, combined with talent and a thorough knowledge of morbid deviation from health and healthy structure, can overcome. The profession at large, and more particularly those who intend to practise in a public service should regard a work of this nature as a valuable present, an occasional reference to which will save them much time and trouble. The cases detailed must be considered authentic, the collection and classification of them demanded a consumption of time and knowledge of the business which few could have brought to it, and even with these advantages, we consider the situation, which we understand Mr. Marshall has held in the recruiting department for some years, to have been an indispensible requisite to the composition of the work. The cases may be regarded as so many beacons, pointing out to the medical officer the difficulties which surround most instances of feigned disability, and especially of bringing the simulators to conviction, a point, which, if possible, should never be left undone. The instances of successful scheming have their value, and can in no manner be considered as at all inculpating the medical officers who had to deal with them. An army surgeon can no more expect to defeat every case of malingering than he can to cure every case of fever, but he must do his best, and it is due from an author to the public to produce some of his bad cases as well as his successful ones.

"To estimate what degree of disease actually exists, and how much of the assumed disability is only pretended, is often attended with considerable difficulty," and we are carefully warned, by the recital of a remarkable case which happened to the late Dr. Gregory of Edinburgh, not to be hasty in making a determination from which we cannot retract. The necessity of proceeding cautiously is further enforced by the following remarks.

"I believe it is admitted, even by the most experienced military medical officers, that notwithstanding every care to draw a just induction from symptoms, we may in some cases form wrong conclusions. In no instance therefore should means be employed, for the purpose of exciting an assumed simulator to return to his duty, which we would regret adopting if the disease were to prove genuine. Restraint and any measure that bears the character of punishment, is not only illegal, but generally inexpedient. But although physical pain, beyond that which may be occasioned by the remedies suitable for the alleged disease, if real, should not be inflicted, a medical officer may in many cases be warranted in endeavouring to produce some mental uneasiness—by abscinding hope, exciting shame, and awakening a sentiment of fear. These means may be employed in a variety of ways; and he who can best employ them, either singly or combined, according to the situation in which he is placed, and the tempers and dispositions of individuals, will be most successful."

We are desirous to give a case or two in proof of what a determined character will endure, and the difficulty which sometimes occurs in convicting an impostor, but, we confess, where there are so many cases in point, it is difficult to make a selection. We take the following of Pat. Maguire, because the case is complete throughout, a perfect conviction ensued, and in the course of his treatment he was under several medical officers of the first respectability.

"Pat. Maguire, a native of Portumna, County Galway, enlisted into the Hon. East India Company's service early in the year 1824. After being a few months at the Depôt, Chatham, he became suddenly affected with loss of power of the right leg and thigh. When standing, he merely touched the ground with the toes, and upon attempting to walk, the limb bent under him. He attributed this feebleness of the leg to a fall he received when descending the barrack stairs; but there was no moral evidence in support of his assertion regarding the fall, and the limb evinced no trace of disease or disability; so that from the first he was suspected of scheming. After being a few months under the care of Dr. Davies, he was transferred to Fort Pitt general hospital, *where he continued for nearly a year, and underwent a great variety of treatment.* Maguire's case must be in the recollection of many of the medical officers who were on duty in Chatham during the years 1824 and 1825. Although still presumed to be an impostor, he was, on the 13th August, 1825, brought before a medical board, who came to the following conclusion: 'The Board have carefully examined Pat. Maguire, and think that he simulates disease to a greater or less degree, but do not consider he is likely to be benefited by further treatment, and therefore recommend him to be discharged from the service.'"

The finding of this Board not having been considered satisfactory at the Horse Guards, he was examined by another medical board on the 28th September, who reported, that they "were of opinion that he (Pat. Maguire) laboured under chronic rheumatism of the right hip; and although they deemed the disease in a great degree simulated, were yet of opinion that he was unfit for the Company's Service, and unlikely to be passed into the service again if discharged."

He was discharged on the 9th October, and in January, 1827, he addressed a letter to Colonel Hay, commanding the Company's Depôt, Chatham, stating that his health was in "a most deplorable situation," and applying for a pension in consequence of having been disabled in the service. In a few days after the date of this letter, he enlisted at Birr, in the 87th regiment, and was brought along with the other recruits from that part of the country to Dublin, where he was at first considered ineligible for the service, on account of the cicatrices of issues which had been established on the loins; but being a country recruit, he was referred to the final decision of a medical board, who approved him. He deserted from the Depôt of the district on the 17th March. When Maguire was examined at Dublin, it was not known that he had been in the Company's Service. On the 15th July of the same year, he, with the view of obtaining a pension, got himself examined at Nenagh by Dr. ———, assistant surgeon to the ——— regiment, who certified, that he was "disabled in consequence of an injury in the loins and right thigh, and that he was incapable of earning a livelihood from the above causes." It is hardly necessary to observe, that this case will afford a useful warning to young medical officers, with regard to the granting of certificates of disability. This document was transmitted to me by a friend of Maguire's, in the hope that I would promote his views, a circumstance which enabled me to ascertain the identity of the invalid from the Company's Service, and the deserter from the 87th regiment. A clue was also obtained as to where he resided, and measures were promptly taken by the recruiting parties, to seize him as a deserter, but without success. It may be observed, that Maguire was employed by a gentleman in the neighbourhood of Portumna, the same person who transmitted the certificate to Dublin, accompanied with a statement asserting, that, "from his own personal knowledge, he (Maguire) had not been able to do any thing for himself since he had been discharged." I think it is highly probable that the certificate granted in July was given at the request of Maguire's employer. It is supposed he has again enlisted, as he has not, since he first absconded, been seen by any of the recruiting parties on duty in the part of the country where he used to reside. The sequel of the case is disclosed in the Appendix page 220, where we are informed that

"Pat. Maguire surrendered himself at this Depôt (Dublin) as a deserter from the 87th regiment, on the 13th of February, 1828. Although there was, from the first, strong presumptive evidence that he was the same person who had been discharged from the Company's Service, still a *doubt* might be entertained in that respect. On examining him, however, every shade of uncertainty was removed. I found him to be the veritable Pat. Maguire that I saw in the East India Company's Depôt Hospital, and the Fort Pitt General Hospital in 1824 and 1825."

The cases of Haddock, page 162, and of Batt, of the Rifle Brigade, 164, are quite as strong, particularly the latter, but our limits will not permit us to copy them. The following is an instance of the successful trade of deceit, which some vagabonds have carried on in the army, and it is a forcible argument to medical and military officers not to be in too much hurry to dispose of a suspected case.

"Last year, a recruit, named Timothy Regan, was discharged from the Company's Service, at Chatham, in consequence of weakness of intellect. A few months after, he was brought before a medical board, at Fort Pitt, for the same defect, he being then a recruit in one of the regiments of the line. It was then discovered that he had, in a very short time, received *six* bounties. He persisted, for a short period, in denying, with great effrontery, that he had enlisted repeatedly; but he at last admitted the charge, and endeavoured to pass the whole business off as a good joke. He was finally ordered to join the 57th regiment, one of the corps in which he had enlisted."

We have been greatly amused, as well as instructed by these pages, but it is time to bring our account of them to a close. The object we have had in view, by these long extracts, is to stimulate our younger brethren in the naval and military services, to think and to enquire; and we can tell them, as the result of long experience, that much of the discipline of any corps or ship they may be called to serve in, will, as well as their own credit, greatly depend on their ability in counteracting feigned disease; and unless they have previously obtained, and industriously preserved a correct knowledge of pathology, their efforts will be vain and frequently grievous. The public have been greatly defrauded by malingerers, as they are technically called, and it is the bounden duty of every medical officer to prevent the continuance of it as far as lies in his power. The utility of the work is not confined to the medical officer only, it is worthy the attention of the military officer also, especially regimental staff officers, and those connected with the recruiting department. The volume may be considered a valuable addition to the military medical library, and as such, we strongly recommend it to the notice of those authorities who preside over the medical departments of the state.
